# Assessment of Uterine Contraction and Atonic Bleeding during the Third Stage of Labor Using Shear Wave Elastography

**DOI:** 10.3390/diagnostics14141490

**Published:** 2024-07-11

**Authors:** Ayumi Okuyama, Junichi Hasegawa, Kohei Seo, Tatsuya Izdebski, Minako Goto, Akihiko Sekizawa, Kiyotake Ichizuka

**Affiliations:** 1 Department of Obstetrics and Gynecology, Showa University Northern Yokohama Hospital, Yokohama 224-8503, Japan; rdtyx182@med.showa-u.ac.jp (A.O.); seo@med.showa-u.ac.jp (K.S.); t.izdebski@med.showa-u.ac.jp (T.I.); minako0607@med.showa-u.ac.jp (M.G.); ichizuka@med.showa-u.ac.jp (K.I.); 2 Department of Obstetrics and Gynecology, Showa University School of Medicine, Tokyo 142-0064, Japan; sekizawa@med.showa-u.ac.jp; 3 Department of Perinatal Developmental Pathophysiology, St. Marianna University Graduate School of Medicine, Kawasaki 216-8511, Japan

**Keywords:** atonic bleeding, uterine contraction, shear wave elastography, obstetric hemorrhage, third stage of labor, gestational age, placenta delivery, hemostasis, transvaginal delivery

## Abstract

Objective: This study aimed to clarify the relationship between fluctuations in uterine stiffness during the third stage of labor and blood loss upon placenta delivery using shear wave elastography. Methods: This prospective cohort study enrolled consecutive singleton pregnant women above 37 weeks of gestation who delivered infants transvaginally at a single perinatal center. Shear wave velocities (SWV) were continuously measured during the third stage of transvaginal labor using transabdominal ultrasound and these values were compared between groups with large (≥500 g) and small amounts of bleeding during this stage. Results: In total, 8 cases of large bleeding and 47 cases of small bleeding were compared. The large amount of bleeding group had a significantly lower median of minimum SWV values (0.97 [0.52–1.01] m/s than the small amount of bleeding group (1.25 [1.04–1.48] m/s *p* = 0.02). However, no significant differences were observed between the two groups in terms of median, mean, and maximum SWV values. The time from delivery of the infant to placental delivery was significantly longer in the large amount of bleeding group (median time: 370.5 s vs. 274 s, *p* < 0.05). Conclusion: Ultrasound quantification of uterine stiffness using shear wave elastography demonstrated that uterine contractions may influence the biological hemostasis of the uterus during the third stage of labor. Baseline uterine stiffness was weak and a longer duration of placental separation might be associated with cases of large amounts of bleeding during this stage.

## 1. Introduction

During pregnancy, the placenta attaches to the decidua layer and receives maternal blood from spiral arteries [1]. However, at the time of delivery, placental separation from the decidua leads to the release of pooled blood into the intervillous space, along with additional bleeding from spiral arteries, resulting in hemorrhage during the third stage of labor.

Biological hemostasis involves the rapid shrinking and stiffening of the uterus, which compresses the spiral arterial lumen at the site of placental implantation [2]. Consequently, postpartum bleeding, predominantly originating from the placental implantation site, could be reduced [1]. 

Shear wave elastography (SWE) is an ultrasonic technique capable of objectively quantifying the stiffness of biological tissues [3]. Shear waves are generated when an acoustic radiation force pulse sequence (ARFI push pulse) from an ultrasound probe applies force on the tissues, propagating through them at relatively low velocities (1–10 m/s) and rapidly attenuating, yet easily detectable using longitudinal ultrasound [3,4]. The shear wave velocity (SWV) increases with tissue stiffness, allowing for an objective assessment [3]. The assessment of uterine stiffness currently relies only on subjective and temporary palpation. Sometimes, there are situations where the uterus is initially assessed as stiff and then the bleeding increases afterward or, conversely, it is assessed as soft and the bleeding does not increase afterward. We hypothesized that myometrial stiffness changes during the third stage of labor and that this physiological change may be associated with blood loss. It is necessary to capture the dynamic changes in uterine contractions over time; hence, we focused on assessing the stiffness of the uterus using SWE. To our knowledge, no previous studies have used SWE to quantify and assess the variability in uterine stiffness.

This study aimed to clarify the relationship between fluctuations in uterine stiffness during the third stage of labor and blood loss upon placenta delivery using SWE.

## 2. Materials and Methods

This prospective cohort study was conducted at Showa University Northern Yokohama Hospital, Japan, between February 2020 and May 2022. SWV was continuously measured using transabdominal ultrasound during the third stage of transvaginal labor. SWV values were compared between groups with large and small amounts of bleeding during this stage.

This study enrolled consecutive singleton pregnant women above 37 weeks of gestation who delivered infants transvaginally at our perinatal center. Cases that had a history of uterine congenital anomalies such as bicornuate uterus, adenomyosis and uterine fibroids located in the anterior wall, or an operative scar in the abdomen were excluded from the present study.

### 2.1. Ultrasound Procedure

**Figure 1:**
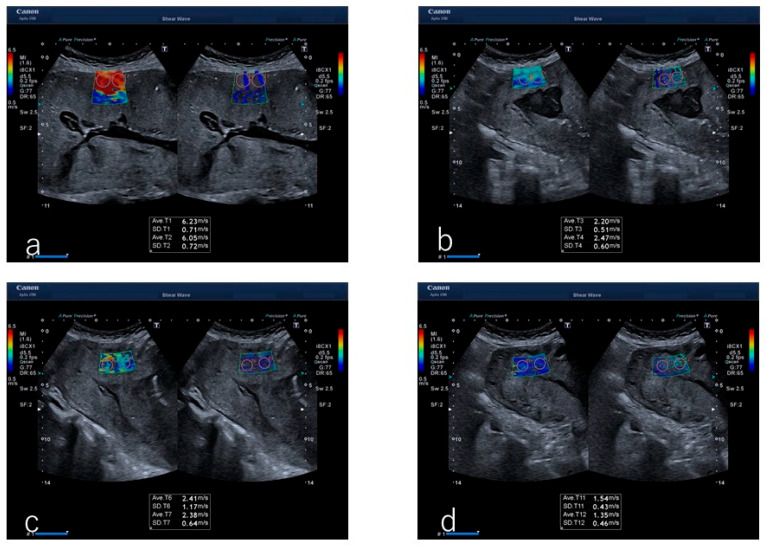
Image of ultrasound shear wave elastography during the third stage of labor in the case of a participant. (**a**) Immediately after infant delivery, (**b**) 240 s later, (**c**) 300 s later, and (**d**) 360 s after infant delivery.

SWE was performed using a 5.5 MHz convex probe (Aplio i700 PVI-475BX, Canon Medical Systems, Otawara, Japan) and analyzed using the manufacturer’s software V5.1. An author (Okuyama) intermittently measured the myometrial SWV transabdominally every 15 s from immediately after infant delivery to placental delivery (Figure 1). The ultrasound transducer was placed at the site between the upper quarter of the anterior wall of the uterus on a straight line connecting the maternal navel and pubic bone for probe placement. Irradiation was performed perpendicular to the anterior uterine wall. The region of interest for SWV measurement was set at 2 × 3 cm or 1 × 3 cm. 

During ultrasound scans, no active management was administered during the third stage of labor, with only umbilical cord clamping performed as part of mixed management. Procedures such as umbilical cord retraction, uterine fundal pressure, or massage were not performed. However, compression hemostasis was continued at the delivery wound. The amount of bleeding during the third stage of labor was measured using gauze, sheet, and suction until complete placental delivery. 

### 2.2. Outcome Measure

Study cohorts were divided into two groups based on the amount of bleeding during the third stage of labor: large (500 g or more) and small (less than 500 g). Various measurements and trends of SWV were compared between these two groups. The clinical characteristics of the individuals were retrospectively collected from medical records.

**Figure 2:**
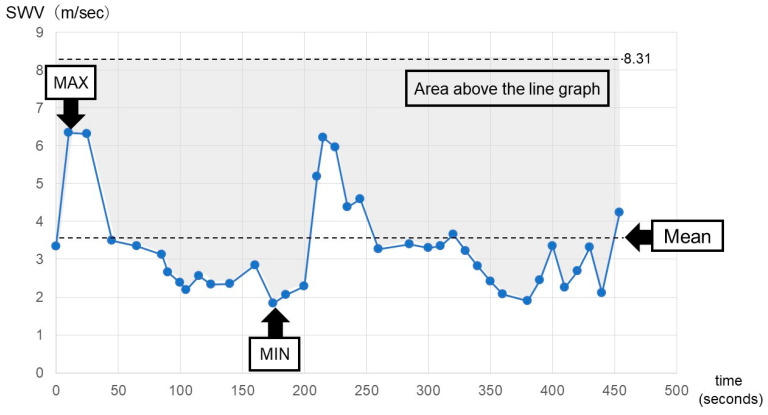
Definition of measured values in shear wave velocity (SWV).

The mean, maximum, and minimum values of SWV were determined for each case. Furthermore, the duration of the third stage of labor and the area above the line graph were calculated (Figure 2). The area above the line graph was obtained by subtracting the area under the plots from the area multiplied by the maximum SWV values among all 55 cases and the duration of the third stage of labor.

### 2.3. Statistical Analysis

Statistical analyses were conducted using the computer statistical software JMP Pro 17 (SAS Institute Inc., Cary, NC, USA). Continuous data were expressed as median (range). The Mann–Whitney U test and Fisher’s exact test were used to analyze variables between groups. Kaplan–Meier plots were used to evaluate comparisons of times between the first, second, and third stages of labor. Statistical significance was set at a *p* < 0.05.

### 2.4. Ethics Statement

All procedures performed in this study involving human participants were in accordance with the ethical standards of the institutional and national research committee and with the 1964 Helsinki Declaration and its later amendments or comparable ethical standards. The Ethics Committee of Showa University, Japan, approved this study (Ethics Approval Number: 19H060). Written informed consent was obtained from all patients. All patient records and information were anonymized and de-identified before the analysis.

## 3. Results

Before commencing this study, the measured intra-observer error was 8.4% (95% confidence interval: 7.3–9.4), calculated using 28 uteruses on the sixth day after delivery.

Fifty-five individuals were analyzed in this study. The large and small amount of bleeding groups included 8 and 47 cases, respectively. Table 1 summarizes the characteristics of these groups. Maternal and neonatal backgrounds did not differ between the two groups. However, the frequency of instrumental delivery was higher (50% vs. 4%, *p* < 0.01) and placental weight was larger (668.5 g vs. 538 g, *p* < 0.01) in a large amount of the bleeding group.

**Figure 3:**
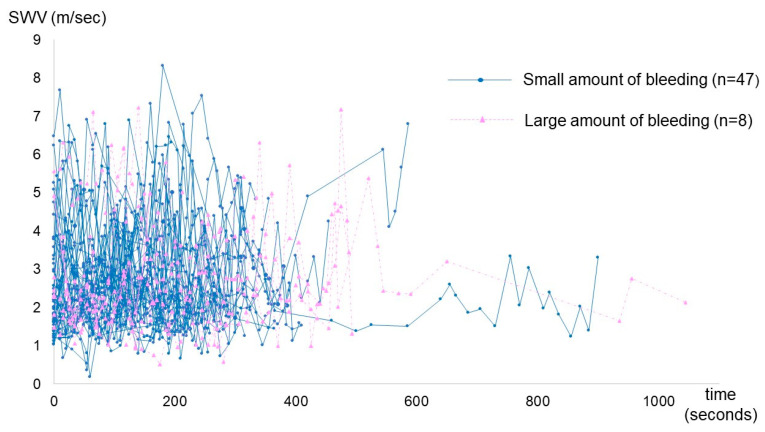
Trend of SWV values during the third stage of labor in each case.

Figure 3 demonstrates the trend of SWV value during the third stage of labor in each case. In total, 1299 SWV data points were acquired via SWE measurements. Fluctuations in uterine SWV during the third stage of labor were observed in each case.

**Figure 4:**
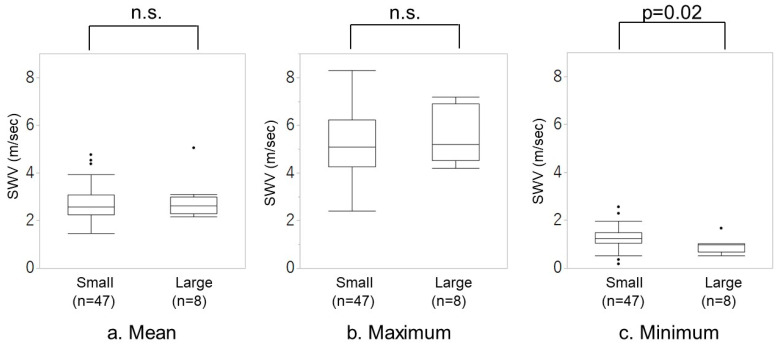
Distribution of SWV values between small and large amounts of bleeding groups. (**a**) Mean value of SWV, (**b**) maximum value of SWV, and (**c**) minimum value of SWV. The top, medium, and bottom of the line in the box represent the 75, 50, and 25% tile, respectively. n.s.: not significant.

Figure 4 illustrates the distribution of SWV values between the small and large amounts of bleeding groups. The large amount of bleeding group had a lower median minimum SWV value (0.97 [0.52–1.01] m/s than the small amount of bleeding group (1.25 [1.04–1.48] m/s). However, no differences were observed in the mean and maximum values of SWV between the two groups.

**Figure 5:**
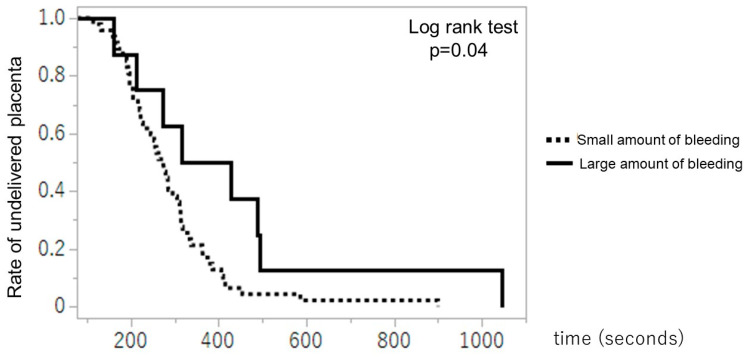
Kaplan–Meier plot for the time from delivery of the infant to the placental delivery between the small and large amounts of bleeding groups.

Figure 5 illustrates the Kaplan–Meier plot for the time from infant delivery to placental delivery between the small and large amounts of bleeding groups. The time from infant delivery to placental delivery was significantly longer in the large amount of bleeding group than in the small amount of bleeding group (median time: 370.5 s vs. 274 s, *p* < 0.05). 

**Figure 6:**
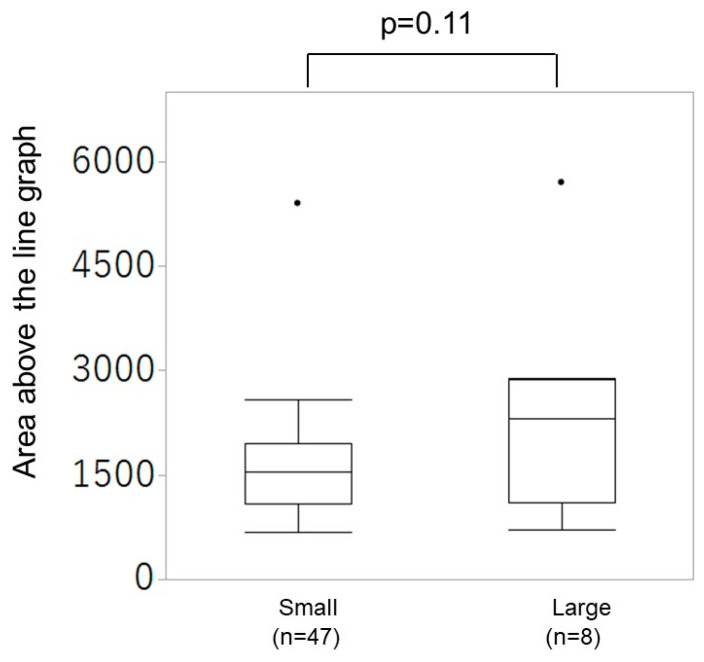
Distribution of area above the line graph between the small and large amounts of bleeding groups.

Figure 6 illustrates the distribution of area above the line graph between the small and large amounts of bleeding groups. The area above the line graph did not differ between the small and large amounts of bleeding groups.

## 4. Discussion

During the third stage of labor, the SWV of the myometrium yielded diverse values. Additionally, the SWV values varied during the third stage of labor within the same case. Our results reveal that the minimum value of SWV was significantly larger in the small amount of bleeding, whereas the mean and maximum value of SWV did not differ. The duration of the third stage of labor was significantly longer in the large amount of bleeding group. However, the area above the line graph during the third stage of labor did not differ.

In this study, the degree of uterine contractions during the third stage of labor was quantitatively evaluated over time. SWE rather than strain elastography was used to evaluate uterine stiffness. Strain elastography is a semi-quantitative evaluation method that calculates an elasticity index by comparing the stiffness of the area being evaluated with the surrounding normal tissue. Furthermore, because external pressure is applied manually, objective evaluation is difficult and there is a possibility of large variability in evaluations. On the other hand, SWE uses the propagation speed of shear waves, so we believe it is possible to perform objective evaluations, especially in the evaluation of organs whose size and shape change. By conducting a quantitative evaluation over time, it was found that there are individual differences in uterine stiffness after delivery and that the uterus continues to contract (harden) and relax (soften) even after delivery. Previous reports have evaluated changes in uterine activity after the third stage of labor using an external tocodynamometer, intrauterine pressure catheters, and electromyography [5,6,7,8]. These reports supported the idea that the uterus repeatedly contracts and relaxes even after the delivery of the infant.

Similar to the findings of previous studies [9,10], our findings revealed that the duration of the third stage of labor was longer in the large amount of bleeding group at the time of placental delivery than in the small amount of bleeding group. Also, uterine contractility and uterine firmness, assessed primarily by palpation, have been considered the physiological mechanism of biological ligation. However, no differences were observed in the mean and maximum values of the SWV. Moreover, the area distribution of the upper part of the line graph in the third stage of labor did not differ between the two groups. Our result only suggested that blood loss is likely greater when the minimum value of uterine stiffness during the third stage of labor is low. It was found that it is not when the uterus becomes stiff that the amount of bleeding is affected but rather when it is at its loosest. It is thought that when the uterus relaxes, pressure on the spiral artery lumen is temporarily released, causing the amount of bleeding to increase. In other words, as stated in the hypothesis, if the uterus is initially assessed as hard but the amount of bleeding subsequently increases, or conversely, if the uterus is assessed as soft but the amount of bleeding subsequently does not increase, this change in stiffness may be related. The results of this study show that the relationship between the uterus after delivery and postpartum hemorrhage is not necessarily determined solely by the absolute stiffness of the uterus. In other words, the stiffer the baseline of the uterus during fluctuations in uterine contractions, the more effectively the biological ligation works.

There are several studies that have shown the effectiveness of using elastography to detect pathological changes in the uterus and placenta [11,12]. It is reported in the previous study that the stiffness of the placenta elasticity index calculated by elastography was increased in FGR cases [11]. In addition, it was demonstrated that as the stiffness of the placenta was increased, detected by SWE in cases with FGR, pre-eclampsia, or GDM, and thus it was concluded that shear wave elastography may be useful for quantifying biomechanical changes in placental tissue and evaluating placental function [12]. There has been a cautious stance worldwide regarding the safety of the fetus during pregnancy. Therefore, this study was planned to use SWE only after the fetal delivery. There have been no reports evaluating uterine contractions and SWE stiffness after delivery and we thought that this could be useful for evaluating stiffness and the function of uterine contractions. 

This study has some limitations. Although SWE requires less measurement skills than those of strain elastography, which applies external pressure manually or mechanically to tissues, the measurements of SWE may depend on experience. In addition, proper probe placement, amount of pressure, and the woman’s position and breathing may also affect the results. It has been demonstrated that reproducibility was better at small depths but that errors become larger at larger depths. In addition, inter-examiner error also increases with depth. Therefore, in order to minimize such errors in the present study, SWE was applied to the upper central 3/4 of the anterior uterine wall. Furthermore, though frequent instrumental deliveries were included in a large number of bleeding groups, instrumental delivery might be needed in cases of weak labor and atonic bleeding. Cases with instrumental delivery might increase bleeding from lacerations; however, this effect is limited because we minimized bleeding from the wound and the amount of bleeding was compared only during the third stage of labor. Nonetheless, we demonstrated subtle fluctuations in the strength of uterine contractions associated with changes in the amount of bleeding, even during the third stage of labor.

## 5. Conclusions

This report is the first to suggest that uterine contractions might affect biological hemostasis of the uterus during the third stage of labor using ultrasound quantitative evaluation of uterine stiffness with SWE. Moreover, baseline uterine stiffness was weak and a longer duration of placenta separation may be associated with cases of large amounts of bleeding during the third stage of labor. As SWE offers a straightforward method for assessing postpartum uterine conditions, it offers valuable insights into the physiology of postpartum uterine contractions. Additionally, SWE can objectively evaluate atonic bleeding, a condition that is challenging to predict based on subjective evaluation alone.

## Figures and Tables

**Table 1:** Characteristics of the participants between the small and large amounts of bleeding groups.

	Blood Loss Small (<500 g) *n* = 47	Large (≧500 g) *n* = 8	*p*-Value
**Maternal**			
Maternal age (y. o)	33 [29–36]	34 [27.25–38.75]	0.60
Primipara	53% (25)	75% (6)	0.44
Height (cm)	159 [156–161]	160 [154.8–165.2]	0.54
Body mass index (kg/m^2^)	20.2 [19.2–22.8]	22.0 [20.7–23.9]	0.17
In vitro fertilization	4% (2)	25% (2)	0.09
Uterine fibroids (posterior wall)	9% (4)	0% (0)	1.00
Gestational diabetes	9% (4)	13% (1)	0.55
Preeclampsia	4% (2)	0% (0)	1.00
Labor induction	38% (18)	75% (6)	0.06
Labor duration			
Total (min)	342 [198–668]	479 [255.8–529.3]	0.60
1st stage (min)	313 [184–540]	329 [149.2–553.5]	0.60
2nd stage (min)	32 [12–49]	88.5 [35.5–179]	0.09
Non-reassuring fetal status	13% (6)	25% (2)	0.32
Instrumental delivery	4% (2)	50% (4)	<0.01
**Neonatal**			
Gestational age	39w5d [38w6d–40w4d]	39w3d [38w5d–41w0d]	0.94
Neonatal Weight (g)	2970 [2804–3316]	3133 [2895.5–3426]	0.37
Placental weight (g)	538 [492–598]	668.5 [622–761.5]	<0.01
Placental abruption	2% (1)	0% (0)	1.00

Values are shown as median and [Interquartile range] or % (*n*).

## Data Availability

Raw data were generated at the Showa University Northern Yokohama Hospital. The derived data supporting the findings of this study are available from the first author, A. Okuyama, upon request.
